# Single Nucleotide Polymorphisms and Insertion/Deletion Variation Analysis of Octoploid and Decaploid Tropical Oil Tea Camellia Populations Based on Whole-Genome Resequencing

**DOI:** 10.3390/plants13212955

**Published:** 2024-10-22

**Authors:** Jiaming Song, Xin Zhao, Bo Lin, Shihui Zhang, Hanggui Lai, Feifei Chen, Dongyi Huang, Jinping Liu, Haiyan Hu, Jian Wang, Wenqiang Wu, Xiaolong Huang

**Affiliations:** 1 Forestry Research Institute, Hainan Academy of Forestry (Hainan Academy of Mangrove), Haikou 571100, China; 15035584294@163.com; 2 School of Breeding and Multiplication (Sanya Institute of Breeding and Multiplication), Hainan Engineering Research Center for Tropical Oil Tea Camellia, Hainan University, Sanya 572000, China; 18092255810@163.com (X.Z.); 15501975073@163.com (S.Z.); hdongyi@hainanu.edu.cn (D.H.); liu3305602@163.com (J.L.); yanhai0987@163.com (H.H.); scoot7@163.com (J.W.); 990818@hainanu.edu.cn (W.W.); hxl2012@163.com (X.H.); 3 School of Tropical Agriculture and Forestry, Hainan University, Danzhou 571700, China; 4 Forest Seed and Seedling General Station of Hainan Province, Haikou 570203, China; 348840031@163.com; 5 School of Life and Health Sciences, Hainan University, Haikou 570228, China

**Keywords:** oil tea camellia, whole-genome resequencing, SNP, variation analysis, genetic diversity

## Abstract

Oil tea camellia (*Camellia* spp.) is an important woody oil crop with a high nutritional and economic value. Whole-genome resequencing (WGR) technology can provide an in-depth understanding of the genetic background of this plant as well as a reference for breeding research, germplasm resource conservation, and genetic modification. In this study, we analyzed SNP and InDel variations in 49 individual oil tea camellia germplasm samples collected from five populations located in three provinces of China: Hainan, Guangdong and Guangxi. The samples were analyzed through WGR after the ploidy of the samples was determined through flow cytometry. A total of 239,441,603 high-quality single nucleotide polymorphisms (SNPs) and 23,510,374 high-quality insertion/deletion variation sites (InDels) were obtained. The distribution of SNPs and InDels in different functional regions differed significantly, with a high density of variations in non-coding regions, such as intergenic regions and introns, and a relatively low density of variations in coding regions. Transition was the main type of SNP variation. A population genetic diversity analysis revealed that the sampled oil tea camellia populations exhibited a high genetic diversity and extensive genetic variation. The genetic diversity of the oil tea camellia populations in the Hainan region was higher than inland regions. This study also determined the genetic diversity of and variations between octoploid and decaploid oil tea camellia in the tropics and between Hainan-based and inland oil tea camellia. Such findings provide a reference for the conservation of germplasm resources and the genetic modification of oil tea camellia.

## 1. Introduction

Oil tea camellia (*Camellia* spp.), also known as “Shanyou”, is a perennial evergreen shrub or small tree that belongs to the family Theaceae and genus *Camellia* L. It is a woody oil crop famous for the high oil content of its seeds [1,2]. This plant has a long history of cultivation in Eastern and Southern Asia, particularly in China, and is an important woody oil cash crop. Oil tea camellia is recognized globally as one of the four major woody oilseed crops together with oil palm (*Elaeis guineensis* Jacq.), olive (*Olea europaea* L.), and coconut (*Cocos nucifera* Linn.). Its advantages include a strong adaptability, drought resistance, and infertile soil resistance [3]. There remains limited research on the specific genetic diversity differences between octoploid and decaploid populations in tropical environments. This study aims to fill this gap by providing insights into how ploidy levels and geographic factors contribute to genetic variation, particularly in the underexplored tropical regions of Hainan. Because of its high economic value, the extensive scope of comprehensive development, a diverse range of applications, and significant utilization potential, it is considered to be a “guaranteed high-yield crop” and “green oil bank” by farmers [4].

Oil tea camellia is primarily distributed in the Yangtze River Basin and southern China but is also grown in small quantities in countries such as Myanmar, Thailand, and Vietnam. In China, oil tea camellia is mainly cultivated in the Hunan, Jiangxi, and Guangxi provinces, with these three regions together accounting for 75.8% of China’s total oil tea camellia cultivation area, with Hunan in particular exhibiting the widest cultivation area accounting for approximately 40% of the total [5]. *Camellia vietnamensis* is particularly suitable for cultivation at low altitudes in the tropical regions of Southern Asia because of its fast growth rate to tall heights and its adaptability to the tropical climate [6]. In the Leizhou Peninsula of the Guangdong Province and from the southeastern part of the Guangxi Zhuang Autonomous Region to Hainan Province, *Camellia vietnamensis* has adapted to climatic conditions and exhibits good flowering and fruiting characteristics. *Camellia vietnamensis* cultivated in Hainan is thought to have originated in Gaozhou City, Guangdong Province; therefore, it is also known as “*Camellia gauchowensis* Chang” in the Hainan region. The history of oil tea camellia cultivation in Hainan may be traced back more than 500 years [7]. A systematic study of the resource distribution of oil tea camellia in Hainan and a field survey revealed that the oil tea camellia germplasm resources in Hainan include wild and artificial cultivars, which are primarily concentrated in nine county-level cities and thirty-eight townships, encompassing approximately 1167.3 hectares [8].

Hainan Island is rich in oil tea camellia resources. The book *Flora of China* indicates that common native oil tea camellia species in Hainan grow in old-growth forests at altitudes above 800 m. According to the *Flora Hainanica*, *A Checklist of Flowering Plants of Islands and Reefs of Hainan and Guangdong Province*, *Hainan Island Crop (Plant) Germplasm Resources Investigation Anthology*, and other sources, Hainan Island contains a wealth of wild common oil tea camellia germplasm resources. Considerable evidence indicates that Hainan Island is the origin of the tropical oil tea camellia and has a long history of oil tea camellia cultivation. In numerous cities and counties in Hainan Province, there exist many oil tea camellia trees that are more than 100 years old, or even several 100 years old, particularly in Ding’an, Chengmai, Tunchang, and Qionghai, where oil tea camellia forests of an older age are preserved. Chengmai County has the highest concentration of oil tea camellia forests, with the largest trees having basal diameters up to 150 cm [8].

Whole-genome sequencing (WGS) is a precise technology that comprehensively reveals an organism’s genetic information. This enables researchers to gain insight into its genetic background and biological functions. Whole genome resequencing (WGR), meanwhile, refers to the sequencing of the genome of a specific individual or population based on an existing reference genome to discover genome-wide variations, such as single nucleotide polymorphisms (SNPs), structural variants, insertion/deletion variation sites (InDels), and copy number variations. These can be used to analyze the molecular genetic characteristics of an individual or population, screen and predict the genes for key economic traits, and study genetic evolution [9].

In January 2022, a research team from the Research Institute of Sub-Forestry, Chinese Academy of Forestry, used diploid oil tea camellia to successfully map the entire genome of oil tea camellia, the results of which clearly illustrated the origin and evolution of oil tea camellia. After more than 4 years of continuous study, this research team successfully obtained a diploid oil tea camellia genome map with a size of approximately 2.95 GB and a Contig N50 of 1.002 MB by applying PacBio third-generation sequencing technology. They accurately localized the genome sequences to 15 chromosomes, achieving a high localization rate of 91.33% [10]. This represents the first high-quality oil tea camellia genome map with chromosome-level precision, setting an example for the assembly of a complex genome of a woody oilseed crop. As of 2023, the whole genomes of some species within the genus *Camellia* of the family Theaceae had been sequenced. Besides oil tea camellia, *Camellia sinensis*, *Camellia japonica*, *Camellia nitidissima*, *Camellia sinensis* var. *pubilimba*, and *Stewartia sinensis* have been sequenced. This is a relatively small number compared with the families and genera of other crops.

At present, some progress has been made in the research of oil tea camellia WGR, which has provided a foundation for evaluating and utilizing oil tea camellia germplasm resources, molecular selective breeding, and the selection of high-quality varieties. oil tea camellia is widely distributed in China, and its genetic diversity is rich across these different geographic environments. Hainan oil tea camellia and that of the inland exhibit different genetic backgrounds and adaptive characteristics due to differences in their geographic location and climate. These differences are likely to be closely related to variations in ploidy level and genome structure. In this study, we selected 49 oil tea camellia germplasm resources from five tropical oil tea camellia populations in Hainan, Guangdong, and Guangxi provinces. After determining the ploidy level of the samples through flow cytometry, we performed WGR analysis to obtain the SNP and InDel variation data from the oil tea camellia samples. Additionally, we used bioinformatics to explore the differences and genetic diversity between octoploid and decaploid oil tea camellia in the tropics and between oil tea camellia in Hainan and the inland. Our findings provide a reference for future studies of oil tea camellia selective breeding and the conservation, rational use, and genetic modification of germplasm resources.

## 2. Results

### 2.1. Sample Collection Information

**Figure 1:**
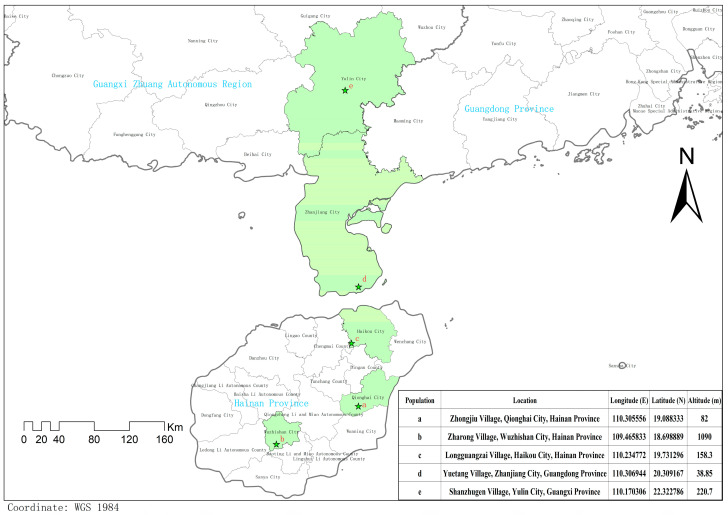
Map of the geographic locations of the oil tea camellia sample collection sites.

The sample populations collected in this experiment were from five different regions. A total of 49 individual plants from these five populations were collected. Populations a, b, c, d, and e were collected from Zhongjiu Village, Qionghai City, Hainan Province; Zharong Village, Wuzhishan City, Hainan Province; Longguangzai Village, Haikou City, Hainan Province; Yuetang Village, Zhanjiang City, Guangdong Province; and Shanzhugen Village, Yulin City, Guangxi Zhuang Autonomous Region, respectively. The names of the sample populations by location are denoted by the population letter codes above in the following text. The information collected regarding the oil tea camellia samples used for statistical analysis is shown in Table 1, and a geographic location map of oil tea camellia sample collection sites is shown in Figure 1.

### 2.2. Results of Oil Tea Camellia Ploidy Determination

**Figure 2:**
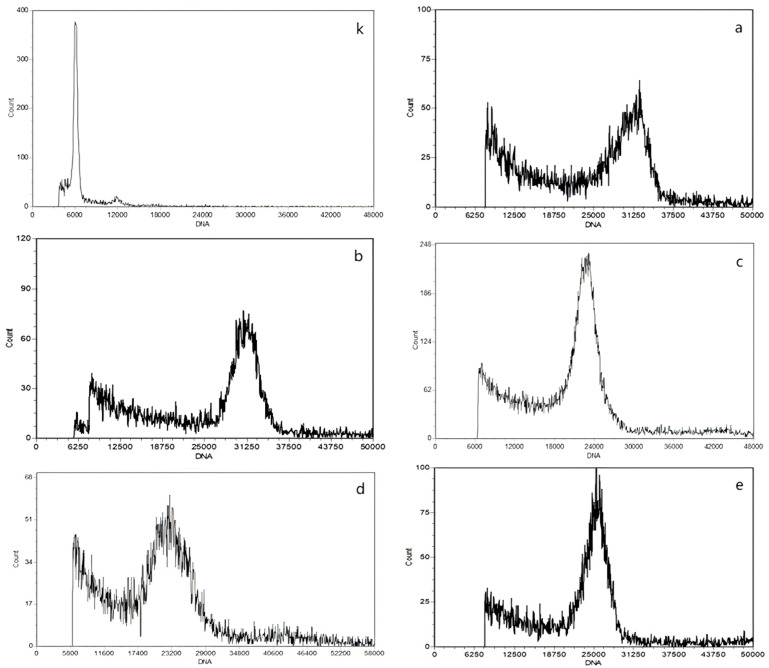
Histograms for the results of the flow cytometry tests on the oil tea camellia samples. Note: (**k**): *Camellia chekiangoleosa* Hu. (control group), (**a**): Zhongjiu No.1, (**b**): Zharong No.7, (**c**): Longguangzai No.1, (**d**): Yuetang No.3, and (**e**): Shanzhugen No.8.

The flow cytometry results for the oil tea camellia samples were obtained after determining the oil tea camellia ploidy levels (Table 2). The histograms obtained through the flow cytometry results are shown in Figure 2. The results indicated that oil tea camellia samples a and b were decaploid, whereas oil tea camellia samples c, d, and e were octaploid. The coefficient of variation of the peak value of each tested sample was within 5%, indicating that the test results are accurate and valid.

### 2.3. Analysis of the WGR Results

#### 2.3.1. Overview of the WGR Data

According to the statistical results (Table 3), the WGR performed on the 49 oil tea camellia samples yielded a total of about 3770 G of raw data. After filtering and processing with the Fastp software, approximately 3722 G of clean data was obtained. Of the paired reads obtained from sequencing, the mean base content was 97.53% for the Q20 quality score and 92.49% for the Q30 quality score, and the mean percentage of the GC base content was 38.76%. These data indicate that the sequencing quality of the samples is good and meets the quality standards of this study. The data can, therefore, be used in subsequent analyses.

The reference genome size used in this study was 2.95 Gb [10]. After aligning the sample genomes with the reference genome, the output (Table 4) indicated that the read alignment rate of all samples ranged between 71.97% and 99.44%, with a mean value of 96.60%. The correct alignment rate of the reads ranged between 61.87% and 85.61%, with a mean value of 76.10%. The mapping quality values of all samples achieved mapQ ≥ 5, indicating that the alignment results were reliable. The above data indicated that the sequencing data were of high quality and exhibited a high alignment with the reference genome. However, the low correct alignment rate of the reads may result from the differences between the reference genome and the sample genome, indicating the presence of a large number of structural variations in the sample genome.

#### 2.3.2. SNP and InDel Variant Analysis

**Figure 3:**
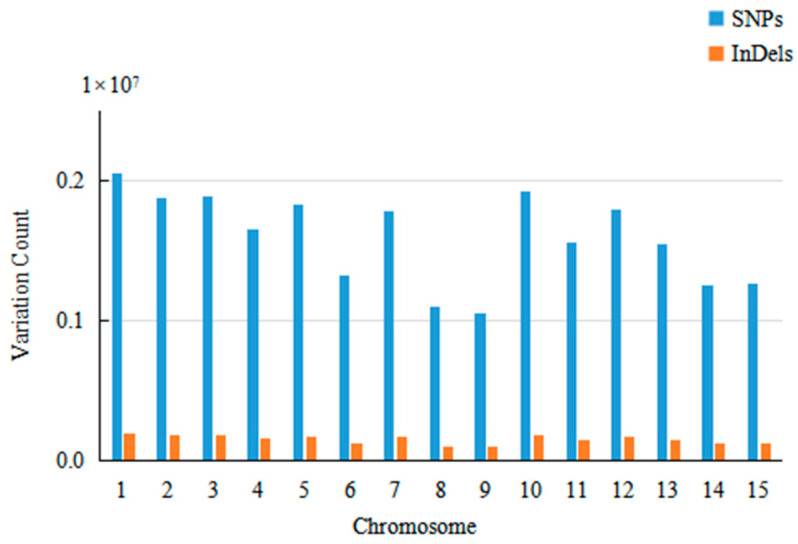
Histogram of the distribution of SNPs and InDel on 15 chromosomes.

After alignment with the reference genome, a total of 247,867,204 SNP markers and 25,687,017 InDel markers were extracted due to the high alignment and sequencing depth. After filtering, the SNP and InDel sites were annotated using the SnpEff software to obtain 239,441,603 high-quality SNPs and 23,510,374 high-quality InDels for the sample population. The distribution of SNPs and InDels for each chromosome is shown in Table 5 and Figure 3. All SNPs and InDels were evenly distributed on the 15 chromosomes of oil tea camellia, and the distribution on each chromosome was approximately uniform.

**Figure 4:**
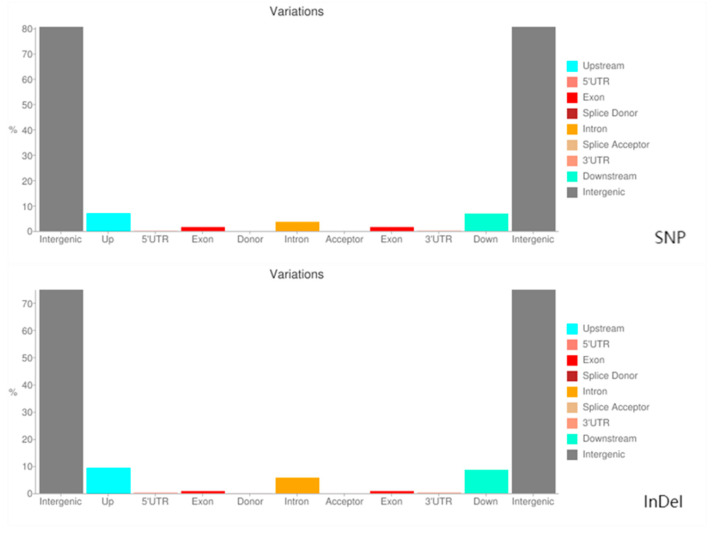
Histogram of the distribution of variation regions for SNPs and InDels.

From the distribution of the SNP and InDel variants in each functional region of the genome (Table 6 and Figure 4), it was evident that the distribution of SNPs and InDels in different functional regions differed significantly. The variation in the sequences of the genomes of the tested samples was primarily concentrated in the non-coding regions, such as intergenic regions and introns. The density of variations in the coding regions was relatively low, which may have occurred because variations in the coding regions were prone to causing deleterious mutations, which were eliminated by strong selection in the evolutionary process.

**Figure 5:**
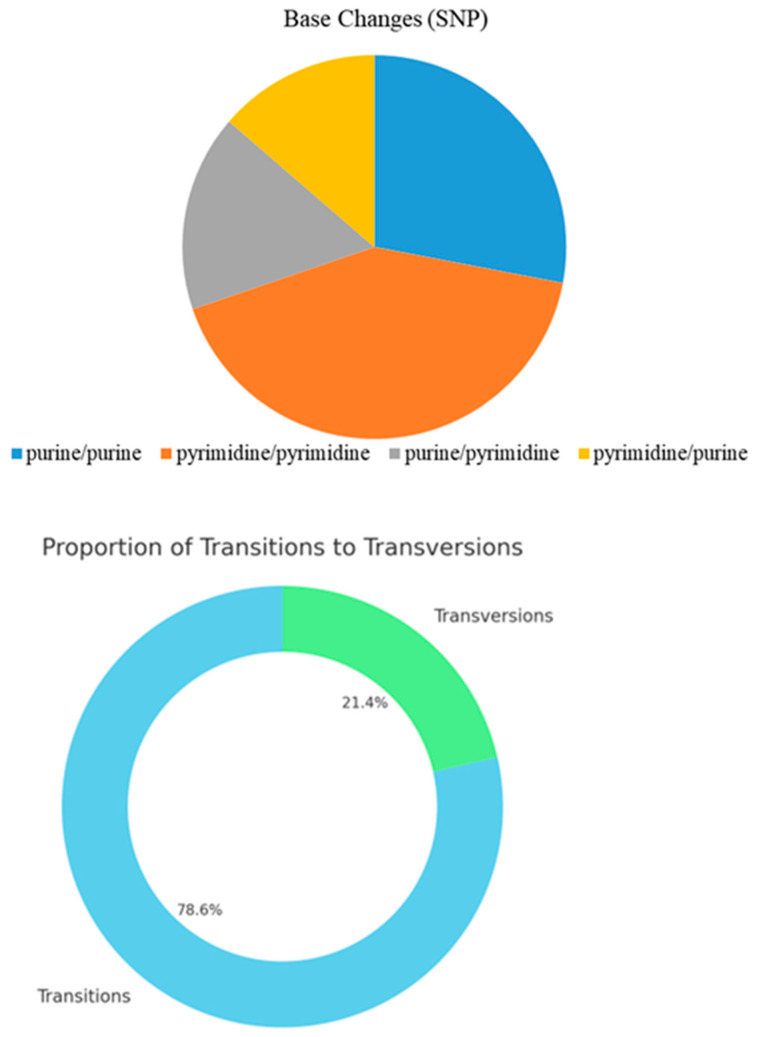
Pie chart (**up**) and circle chart (**down**) of the number and proportion of transition and transversion mutations.

By analyzing the types of base mutations at the SNP sites, the results (Table 7, Figure 5) indicated that the mean Ts/Tv value was 3.672. In this sample genome, transitions were the dominant type of SNP variation, a result that is consistent with the mutational pattern in most species, whereby transitions are more likely to occur and be retained compared with transversions because of complementary base pairing constraints.

Based on their effect on gene function, the variations were categorized into the following four types: HIGH, LOW, MODERATE, and MODIFIER. The results (Table 8) indicated that the vast majority of SNP and InDel variations were MODIFIER types (i.e., those located in non-coding regions), which accounted for 98.345% and 99.091% of the total number of variations, respectively, which is consistent with the above results. This suggests that although the number of variations in the oil tea camellia genome is large, most of them may not directly alter gene function, and only a small number were categorized as HIGH, accounting for 0.063% of the SNPs and 0.606% of the InDels. The latter type will significantly alter gene function by introducing premature stop codons and disrupting intronic splice sites. Although these variations are few in number, they may contain important adaptive loci or domestication genes. MODERATE effect variations accounted for 0.921% and 0.217% of the SNPs and InDels, respectively, and were primarily amino acid substitutions, which may also alter protein function. In contrast, although LOW effect variations changed codons, they were mostly synonymous mutations with less impact on gene function and accounted for lower proportions of the SNPs and InDels.

#### 2.3.3. Genetic Diversity Analysis

**Figure 6:**
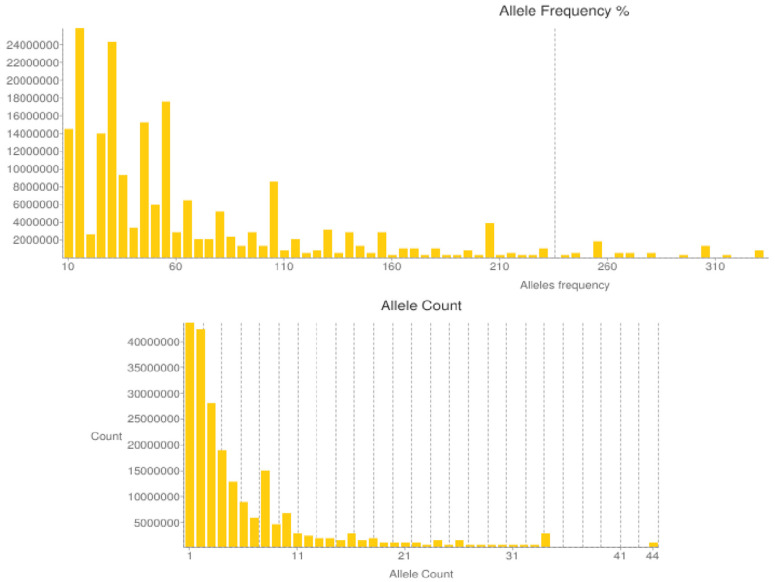
Histogram of the distribution of SNP allele frequency percentages (**top**) and counts (**bottom**).

**Figure 7:**
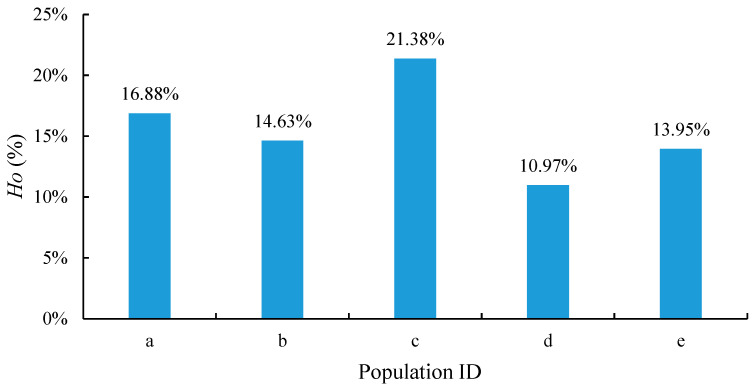
Histogram for the heterozygosity of the five oil tea camellia populations (*Ho*).

The SNP allele frequency percentage and count graphs are shown in Figure 6. From the allele frequency distribution graph, it was evident that the frequencies of most of the alleles were low, primarily concentrated around the 10% frequency interval. The low number of alleles with a high frequency indicates that only a small number of alleles were very common in this population. The presence of a large number of low-frequency alleles also indicated that there was a high genetic diversity and extensive genetic variation in this oil tea camellia population. The number of alleles with a count of one was the highest, which may indicate that these oil tea camellia populations have recently produced alleles or experienced higher mutation rates, providing rich genetic resources for the oil tea camellia populations. The heterozygosity (*Ho*) of the five oil tea camellia populations was 16.88%, 14.63%, 21.38%, 10.97%, and 13.95%, respectively (Figure 7), with the largest *Ho* value recorded in population c. A high heterozygosity is usually associated with a good adaptive capacity and survival potential, suggesting that this population is genetically more diverse.

## 3. Discussion

WGR technology may be used to obtain information on genome-wide locus variation. It has been widely used in gene localization, genetic map construction, mutation site identification, and genetic evolution research [9,11]. Genetic diversity plays a crucial role in the evolutionary capacity of species; those with a higher genetic diversity tend to adapt more efficiently to changes in the environment, whereas those with a lower genetic diversity may gradually lose their competitiveness and become less adaptable through long-term natural selection [12].

In the present study, we used genome-wide allele frequencies, the heterozygosity (*Ho*) of oil tea camellia, and the diversity indices of phenotypic traits to assess genetic diversity. A high heterozygosity is usually associated with a good adaptive capacity and survival potential. The results indicated the presence of a large number of low-frequency alleles in the sample genomes, which suggests a high genetic diversity and extensive genetic variation in the oil tea camellia populations. Of the five oil tea camellia populations in the sample genome, population c achieved the largest *Ho* value, indicating a higher genetic diversity among this population compared with the others. Populations a and b were more genetically diverse than d and e. The high genetic diversity observed in oil tea camellia populations is consistent with the findings of Ye et al. [13], who highlighted significant genetic diversity across various Camellia populations, partly attributing this diversity to environmental adaptation and ploidy-level variation. This rich genetic diversity provides a foundation for breeding programs targeting traits like ecological adaptation, oil yield, genetic modification, and biodiversity conservation of the species.

In addition to its role in breeding and adaptation, maintaining a high genetic diversity is crucial for preserving the beneficial bioactive compounds present in oil tea camellia, which are valuable for medicinal and commercial applications. Teixeira and Sousa [14] emphasized the diverse biological activities of Camellia species, including their antioxidant, anti-inflammatory, and anti-cancer properties, which are linked to the genetic variability of these plants. Additionally, Li et al. [15] further elaborated on the therapeutic benefits of camellia oil, highlighting its positive effects on cardiovascular health due to its rich unsaturated fatty acid content. Therefore, the diversity observed in our study is instrumental in enhancing these health-promoting compounds, and conservation efforts should focus on maintaining this genetic diversity to maximize both the medicinal and economic value of oil tea camellia.

The above findings not only enhance our understanding of the oil tea camellia genome, but also provide a basis for the conservation of its germplasm resources and genetic modification. However, it is important to consider ethical issues related to genetic modification. While our study provides a foundation for potential genetic modifications, any practical applications should undergo careful evaluation regarding their ecological impacts and ethical implications. Future studies may further explore how ploidy-level and environmental factors specifically affect the genetic structure and gene expression of oil tea camellia and how these differences influence the survival and reproduction strategies and the evolutionary direction of oil tea camellia. However, some of the observed differences could not be explained in this study because of the large number of genomes associated with the octoploid and decaploid oil tea camellia samples. This was also affected by the presence of a large number of DNA repeated sequences, high heterozygosity, genome complexity, and detection errors in the sequencing methods and technology. In the future, sequencing methodology and technology improvements are needed to further reduce detection errors.

The WGR study has increased our understanding of the genetic diversity of oil tea camellia through variation detection and population genetic analysis. Our findings provide strong support for the discovery of high-quality alleles and the genetic analysis of important agronomic traits. Additionally, the resequencing data may be used to identify various molecular markers, construct high-density genetic maps, and accelerate the molecular breeding process of oil tea camellia. However, there are still important issues to be solved in terms of the WGR of oil tea camellia, such as the relatively high sequencing cost and major challenges such as the huge amount of genome data, the numerous repeated sequences, and the extremely complex homologous and heterologous structures of the various subgenomes of oil tea camellia. Additionally, data analysis and bioinformatics processing also pose challenges, and the validation of and functional research into the various sites must be strengthened. With the increasing maturity of sequencing technology and the continuous improvement of bioinformatics analysis, more breakthroughs are bound to be achieved in future research on the WGR of oil tea camellia.

## 4. Materials and Methods

### 4.1. Sample Collection

The samples collected in this study were selected from live oil tea camellia tree clusters over 50 years of age in Hainan Province and inland China (Guangdong and Guangxi provinces). When sampling, 5–6 fresh young leaves (preferably with newly grown red young leaves at the top of the oil tea camellia plant) were selected from each plant, placed into a sealed bag, and numbered. Next, the sealed bags were placed in dry ice for storage and used to determine the ploidy level through flow cytometry and WGR analysis.

### 4.2. Determination of Sample Ploidy Levels and Genomic DNA Extraction

Flow cytometry was performed [16] along with the cetyltrimethylammonium bromide (CTAB) method [17,18]. The calculation formula was as follows: Genome size of the test sample = control genome size × fluorescence intensity of the test sample ÷ control fluorescence intensity [19,20].

### 4.3. Resequencing Data Acquisition and Processing

#### 4.3.1. WGR Library Construction

The extracted DNA samples were sent to Wuhan Benagen Technology Co., Ltd., located in Wuhan City, Hubei Province, China, for sequencing to obtain raw WGR data. The sequencing platform used was BGI (BGI-Shenzhen, China), the sequencing library used was DNBSEQ, and the sequencing method was paired-end sequencing.

#### 4.3.2. Raw Data Quality Control (QC)

The initial fluorescence image files acquired from the sequencing platform were converted into raw data. These data were subject to QC through filtering to remove adapter sequences, low-quality bases, and unrecognizable nucleotides (N). The Fastp (version 0.20.0, developed by OpenGene, Shenzhen, China) software [21] was used for processing to remove low-quality sequences. Through filtering, higher-quality data (i.e., clean data) was obtained and statistically analyzed in terms of the number of reads, Q20 and Q30 quality scores, and GC content.

#### 4.3.3. Alignment of Sequencing Data to a Reference Genome

Clean reads were aligned and sequenced against the reference genome sequence using BWA (version 0.7.17, developed by Heng Li, Broad Institute, Cambridge, MA, USA) [22], SAMTOOLS (version 1.6, originally developed by the Sanger Institute, Hinxton, Cambridgeshire, UK) [23], and GATK (version 4.3.3.0, developed by the Broad Institute, Cambridge, MA, USA) [24] software. The reference genome was the diploid oil tea camellia sequence published in the NCBI database (https://www.ncbi.nlm.nih.gov/datasets/genome/GCA_022316695.1, accessed on 1 July 2023) [10]. Statistical analysis was performed after obtaining the alignment information.

#### 4.3.4. Variation Detection and Filtering

Sequence files were polymerase chain reaction-labeled for repeated sequences using the MarkDuplicates tool in the GATK software. These files were indexed using the index tool in the SAMTOOLS software. Variation was detected using the GATK software [24,25].

#### 4.3.5. Variation Annotations

The SnpEff (version 5.2, developed by Pablo Cingolani, La Jolla, CA, USA) software [26,27] and the gff annotation file of the oil tea camellia reference genome (https://github.com/Hengfu-Yin/CON_genome_data, accessed on 1 April 2024) were used to annotate variations in the SNP and InDel sites in the filtered VCF file. The locations and types of variations were also obtained.

## 5. Conclusions

In this study, through WGR of 49 tropical oil tea camellia germplasm resources, 247,867,204 SNPs and 25,687,017 InDel variation sites were obtained with 96.60% alignment with the reference genome. After filtering out low-quality reads and excluding heterozygous and missing data, we obtained 239,441,603 high-quality SNPs and 23,510,374 high-quality InDels. A variation analysis of 49 oil tea camellia samples using these SNP and InDel loci, which cover the whole genome, revealed that there were significant differences in the distribution of SNPs and InDels in various functional regions, with a higher distribution in the intergenic and upstream regions. This suggests that there is greater evolutionary variability in these regions, and the SNP and InDel variations in the non-coding regions may have a significant impact on oil tea camellia gene expression regulation and genetic diversity. In contrast, variations in the genomic sequences of the tested samples were low in the coding regions. This may have occurred because the variation in coding regions tends to cause deleterious mutations, which are eliminated by strong selection during evolution. Based on our analysis of base mutations at SNP sites, we found that transitions were the predominant type of SNP variation in the genome of this sample. This finding aligns with the typical mutation pattern in most species, where transitions are more likely to occur and be retained than transversions due to complementary base pairing constraints.

The results of this study provide valuable insights into the genetic diversity and variation patterns of oil tea camellia, particularly in tropical populations. The high-quality SNP and InDel datasets generated here offer a valuable resource for future research, including the identification of key genetic markers for breeding programs aimed at improving ecological adaptability, oil yield, and resistance to environmental stresses. Furthermore, our findings highlight the importance of non-coding regions in maintaining genetic diversity, suggesting that future studies should focus on the functional impact of variations in these regions, especially their role in gene regulation and adaptation mechanisms. Advances in sequencing technologies and bioinformatics approaches will be essential for further reducing detection errors and enhancing the understanding of the complex genomic architecture of oil tea camellia. Such efforts will contribute significantly to the conservation and genetic improvement of this economically important species.

## Figures and Tables

**Table 1:** Oil tea camellia sample collection information.

Population ID	Sampling Location	Longitude (E)	Latitude (N)	Altitude (m)	Age of Central Plant	Soil Type	Habitat	Mean Ground Diameter/cm	Maximum Ground Diameter/cm	Number of Samples
a	Zhongjiu Village, Qionghai City, Hainan Province	110°18′	19°05′	82	590 years	Laterite	Shrub forest	25	60	4
b	Zharong Village, Wuzhishan City, Hainan Province	109°27′	18°41′	1090	Over 100 years	Lateritic red earth	Tropical rainforest	20	58	10
c	Longguangzai Village, Haikou City, Hainan Province	110°14′	19°43′	158.3	60 years	Laterite	Farm	20	42	10
d	Yuetang Village, Zhanjiang City, Guangdong Province	110°18′	20°18′	38.85	60 years	Torrid red soil	Field	15	45	13
e	Shanzhugen Village, Yulin City, Guangxi Zhuang Autonomous Region	110°10′	22°19′	220.7	50 years	Lateritic red earth	Shrub forest	15	42	12
Total										49

**Table 2:** Flow cytometry results of the oil tea camellia samples.

Sample Population	Sample Name	Peak Value	Coefficient of Variation	Genome Size/Gb	Ploidy
k	*Camellia chekiangoleosa* Hu. (Control Group)	6098	3.43	2.73	2X
a	Zhongjiu No.1	31,398	3.88	13.1586	10X
b	Zharong No.7	31,100	3.01	13.0221	10X
c	Longguangzai No.1	21,998	4.78	10.2482	8X
d	Yuetang No.3	23,318	4.13	10.5764	8X
e	Shanzhugen No.8	25,588	3.37	10.7289	8X

**Table 3:** Sequencing data quality.

No.	Q20 (%)	Q30 (%)	GC Content	No.	Q20 (%)	Q30 (%)	GC Content
ZJ01	97.85	93.15	37.53	YT02	96.12	87.93	37.34
ZJ02	96.98	90.22	37.56	YT03	97.45	92.00	37.34
ZJ03	97.89	93.26	37.48	YT04	97.57	91.75	36.37
ZJ05	97.87	93.17	37.82	YT06	96.54	89.21	36.89
ZR01	97.77	93.90	42.78	YT07	97.42	91.93	37.19
ZR02	98.31	95.35	42.16	YT08	97.31	91.61	37.15
ZR03	97.76	93.95	40.70	YT09	96.84	89.88	36.90
ZR04	98.28	95.28	41.94	YT10	96.81	90.14	36.86
ZR05	98.25	95.17	41.75	YT11	96.60	89.39	36.88
ZR06	98.37	95.58	39.90	YT12	96.61	89.45	36.98
ZR07	98.02	94.58	41.59	YT14	97.46	92.06	36.95
ZR08	97.84	94.14	42.03	YT15	97.07	90.82	37.15
ZR09	98.25	95.27	45.78	SZG01	96.82	89.90	37.47
ZR10	98.19	95.09	40.61	SZG02	96.58	89.62	37.86
LGZ01	98.32	95.52	39.61	SZG03	97.15	91.31	38.01
LGZ02	97.90	94.44	39.36	SZG04	96.77	90.19	37.86
LGZ03	98.12	95.02	39.38	SZG05	96.32	88.41	36.91
LGZ04	97.99	95.30	38.71	SZG06	96.15	87.75	38.62
LGZ05	97.91	94.64	39.25	SZG07	97.24	91.09	38.79
LGZ06	98.50	95.42	39.19	SZG08	98.62	94.81	37.75
LGZ07	98.12	94.97	39.46	SZG09	96.68	89.19	38.67
LGZ08	97.99	95.32	39.01	SZG10	98.37	93.83	37.70
LGZ09	98.40	95.68	39.36	SZG11	97.20	90.56	38.89
LGZ10	98.41	95.10	38.95	SZG12	97.16	90.35	37.39
YT01	96.62	89.44	37.24	Mean	97.53	92.49	38.76

**Table 4:** Alignment data statistics.

No.	Alignment Rate (%)	Correct Alignment Rate (%)	No.	Alignment Rate (%)	Correct Alignment Rate (%)
ZJ01	99.31	66.47	YT02	98.56	76.47
ZJ02	92.96	70.71	YT03	99.22	73.33
ZJ03	97.82	75.90	YT04	99.21	75.62
ZJ05	96.96	72.65	YT06	98.89	76.32
ZR01	94.02	75.29	YT07	99.11	77.40
ZR02	95.80	79.89	YT08	99.15	73.94
ZR03	96.13	78.90	YT09	98.96	77.05
ZR04	97.92	81.87	YT10	99.26	72.85
ZR05	97.16	81.18	YT11	99.18	74.81
ZR06	95.28	82.11	YT12	98.94	75.04
ZR07	94.62	77.91	YT14	99.43	75.41
ZR08	81.89	67.03	YT15	98.78	74.90
ZR09	71.97	61.87	SZG01	96.81	73.83
ZR10	91.21	76.66	SZG02	98.32	73.58
LGZ01	99.44	85.61	SZG03	91.60	69.05
LGZ02	99.15	82.42	SZG04	97.94	73.66
LGZ03	99.16	82.60	SZG05	96.13	70.47
LGZ04	99.03	81.35	SZG06	98.81	74.68
LGZ05	99.35	82.66	SZG07	97.34	78.85
LGZ06	99.34	81.68	SZG08	97.77	75.49
LGZ07	99.22	83.24	SZG09	93.88	70.38
LGZ08	99.05	83.10	SZG10	97.24	74.43
LGZ09	99.14	84.24	SZG11	88.55	64.49
LGZ10	98.82	80.85	SZG12	97.19	72.64
YT01	98.52	78.23	Mean	96.60	76.10

**Table 5:** Distribution of SNPs and InDels on 15 chromosomes.

Chromosome	SNPs	InDels	Chromosome	SNPs	InDels
1	20,518,846	2,030,755	9	10,568,505	1,025,902
2	18,765,410	1,850,573	10	19,286,835	1,885,013
3	18,863,375	1,904,873	11	15,649,862	1,506,655
4	16,548,516	1,620,970	12	17,933,417	1,761,125
5	18,279,754	1,814,329	13	15,543,295	1,482,437
6	13,300,085	1,329,549	14	12,550,212	1,250,940
7	17,848,819	1,729,788	15	12,726,230	1,228,466
8	11,058,442	1,088,999	Total	239,441,603	23,510,374

**Table 6:** Distribution of SNPs and InDels in each genomic region.

Type (Alphabetical Order)	SNP		InDel	
	Count	Percent	Count	Percent
DOWNSTREAM	19,496,920	6.922%	2,540,421	8.687%
EXON	4,410,824	1.566%	233,871	0.8%
INTERGENIC	226,919,623	80.561%	21,897,761	74.88%
INTRON	10,261,566	3.643%	1,686,222	5.766%
SPLICE_SITE_ACCEPTOR	19,329	0.007%	4362	0.015%
SPLICE_SITE_DONOR	15,664	0.006%	2720	0.009%
SPLICE_SITE_REGION	176,089	0.063%	25,270	0.086%
UPSTREAM	19,697,409	6.993%	2,719,777	9.3%
UTR_3_PRIME	397,423	0.141%	70,031	0.239%
UTR_5_PRIME	280,884	0.1%	59,586	0.204%
Missense Variant	2,594,175	0.92%	167,636	0.57%
Genome Total Length	2,891,061,056			
Genome Effective Length	2,640,572,740			
Variant Rate	1 variant every 11 bases		1 variant every 112 bases	

**Table 7:** SNP base mutations.

Base Changes	Transitions (Ts)		Transversions (Tv)	
	A	G	C	T
A	0	29,499,406	6,892,135	9,304,074
G	58,391,906	0	5,606,594	10,056,400
C	9,977,366	5,595,200	0	58,363,346
T	9,366,922	6,880,418	29,507,836	0
Total	1,103,905,690		300,594,047	
Ts/Tv	3.672			

**Table 8:** Number and proportion of SNPs and InDels at different impact levels.

Type (Alphabetical Order)	SNP		InDel	
	Quantity	Ratio	Quantity	Ratio
HIGH	176,782	0.063%	177,132	0.606%
LOW	1,891,522	0.672%	25,270	0.086%
MODERATE	2,594,175	0.921%	63,552	0.217%
MODIFIER	277,013,252	98.345%	28,977,751	99.091%

## Data Availability

Data are available on request from the corresponding authors.
